# Social Perception of Risk-Taking Willingness as a Function of Expressions of Emotions

**DOI:** 10.3389/fpsyg.2021.655314

**Published:** 2021-06-01

**Authors:** Shlomo Hareli, Shimon Elkabetz, Yaniv Hanoch, Ursula Hess

**Affiliations:** ^1^The Laboratory for the Study of Social Perception of Emotions, Department of Business Administration, University of Haifa, Haifa, Israel; ^2^Southampton Business School, University of Southampton, Highfield, Southampton, United Kingdom; ^3^Department of Psychology, Humboldt-University of Berlin, Berlin, Germany

**Keywords:** emotion expression, risk taking, social perception, risk domain, person perception

## Abstract

Two studies showed that emotion expressions serve as cues to the expresser’s willingness to take risks in general, as well as in five risk domains (ethical, financial, health and safety, recreational, and social). Emotion expressions did not have a uniform effect on risk estimates across risk domains. Rather, these effects fit behavioral intentions associated with each emotion. Thus, anger expressions were related to ethical and social risks. Sadness reduced perceived willingness to take financial (Study 1 only), recreational, and social risks. Happiness reduced perceived willingness to take ethical and health/safety risks relative to neutrality. Disgust expressions increased the perceived likelihood of taking a social risk. Finally, neutrality increased the perceived willingness to engage in risky behavior in general. Overall, these results suggest that observers use their naïve understanding of the meaning of emotions to infer how likely an expresser is to engage in risky behavior.

## Introduction

Occasionally a person may need to estimate the extent to which another individual is willing to take a risk. This is the case, for example, when a financial advisor is asked to make a recommendation for a prospective investor, when a doctor advises a patient about possible treatments or when negotiators attempt to figure out what offer they could put on the table without their counterparts backing off. One important question that arises in such a situation is how observers can estimate or judge the risk proneness of another person, especially when that person is unknown to them.

Relatively few studies have examined this question, but two sources of information have been isolated. First, a person’s own risk proneness serves as an anchor for judging other people’s risk proneness (e.g., Hsee and Weber, 1997; Chakravarty et al., 2011). A second source of information is stereotype information related to the social identity of the other person such as their age (Rosen and Jerdee, 1976) or gender (Siegrist et al., 2002). In the present research, we suggest that observers might use a third cue for their judgment: the other’s emotional state as reflected by their emotional expression.

According to appraisal theories of emotion, specific emotions are differentiated by their pattern of appraisals (e.g., Frijda, 1986; Scherer, 1987; Lazarus, 1991). Emotions are responses to major concerns of the individual (Frijda, 1986) and prepare the individual to respond appropriately to the emotion eliciting event (Frijda, 1988). This implies that an appraisal pattern associated with a specific emotion is also associated with a specific action tendency or action readiness. These are behaviors that address the issue that gave rise to the emotion in the first place (Frijda, 1986; Scherer, 2005). For example, fear is associated with tendencies to engage in protective behavior, often in flight. By contrast, anger is linked with a tendency to move against, to oppose the source of the anger (Frijda et al., 1989). Thus, specific emotions are associated both with specific appraisals as well as specific behaviors (Roseman et al., 1994).

People are aware of the link between emotions, appraisals, and action tendencies and can use this awareness to deduce from emotional expressions how the expresser appraises the situation and what they are likely to do next (Hareli and Hess, 2019). For example, if someone shows fear in reaction to an event, protective behavior is a likely action tendency. That is, emotional expressions can serve as cues to others about the expresser’s behavioral intentions and motives (Fridlund, 1994), and indicate an intention to act in a specific way (Scarantino, 2017).

Therefore, in a given context, the interactants’ emotion expressions should limit the range of likely actions to those that are congruent with the underlying action tendency. For example, when someone shows anger, an observer may assume (a) that this person is unlikely to behave in ways that are not associated with anger, such as staying away or remaining passive, and (b) that this person is more likely to behave in ways that are associated with the behavior typical of the emotion, such as acting against someone else.

The present research focuses on inferences regarding potential risk-taking by an emoter. Specifically, we consider the effect of knowing that a person is angry or sad on the perceived risk proneness of the expresser. Anger is associated with a tendency to move against its object and sadness with a tendency to remain inactive. In a given context, it may not be actually the case that someone can move against someone else or have the option to be inactive, rather, the underlying action tendencies will play out in ways appropriate to the context. Thus, in a risk-taking context, anger and sadness should be indicative of the risk-taking willingness of the emoter in ways that are suitable to that context.

For example, since an angry person signals a willingness to move against the anger-eliciting object, something that is likely to involve a risk, anger may be seen as an indication that the individual is prepared to engage in other risks as well. By contrast, because sadness is associated with a tendency towards inaction, this may be taken as a more general sign that the emoter will avoid risky action.

People’s preference to take risks varies across different life domains (Soane and Chmiel, 2005; Blais and Weber, 2006; Hanoch et al., 2006). That is individuals do not appear to be consistently risk seeking or risk averse across different domains and situations. For example, Wang et al. (2009) looked at the effect of birth status on risk-taking propensity in different domains. They found that those who were born last in the family were more likely to take a risk involving an environmental challenge such as exploring a new place than their older siblings. By contrast, they were as likely as their older siblings to take a risk involving competition with others such as taking a leading role in one’s group. Perceived willingness to take risks may also be expected to be domain specific and be differentially determined by the type of emotion perceived.

As mentioned above, observers associate emotions with specific action tendencies (Frijda, 1986). For example, moving against another is more typical of an angry person than a happy one (Frijda et al., 1989). By contrast, happiness is associated with a tendency or urge to play and broaden one’s experience (Fredrickson, 2004). Hence, observers are more likely to assume that someone who is angry will act aggressively than in a playful way. Aggression involves a risk in the social and ethical domains. Any act in the recreational domain, which in many cases involves some playful spirit, is less likely for an angry person. Accordingly, taking a risk in this domain also may seem less plausible. By contrast, it is plausible that a happy person is perceived to be more likely to take a risk in a domain associated with play, for example, by engaging in a more dangerous recreational activity such as sky diving, but not necessarily to take a financial risk on the stock market or taking a risk involving ethical issues.

We report the results of two studies testing (a) the notion that emotion expressions can be used as cues to risk taking and (b) that different emotions are indicative of different types of risk taking.

## Study 1

To test the idea that people use others’ emotional expressions as cues to risk proneness, participants saw a photo of a man or a woman expressing either sadness, anger, or neutrality (as a control condition). Participants were told that the photo depicts how the person reacted to a specific event and therefore represents the person’s current state of mind. Participants were then asked to assess the emoter’s willingness to take risks in five risk domains: ethical, financial, health and safety, recreational, and social (Blais and Weber, 2006). We focused on expressions of anger and sadness since, as noted above, these two emotions are associated with different action tendencies that are relevant to risk taking. We predicted that an angry person will be perceived as more likely to engage in risky behavior than a sad person. We included a neutral expression as a control condition to assess if any effects found are due to increased perceived risk-taking for anger or decreased perceived risk-taking for sadness or both. Based on previous research, we also expected that, regardless of the type of emotion shown, men would seem more likely to take risks than women due to gender stereotypes related to risk taking (Siegrist et al., 2002).

### Method

#### Participants

In total, 224 (120 women) participants with a mean age of 42 years (SD = 12.88) who were recruited through Amazon MTurk completed the study and passed control questions probing for attention. Based on a sensitivity analysis using G*Power (Faul et al., 2007), given our sample size, the minimum effect size that the experiment had 80% power to detect was *f* =0.21.

#### Stimulus Materials

Facial expressions of anger, sadness, and neutrality by four young men and women were taken from the FACES database of facial expressions (Ebner et al., 2010).

#### Procedure and Dependent Measures

After consent was obtained, participants were told that taking risks is very common and that people engage in different types of risk-taking activities daily. Thus, whereas some people take many risks, others prefer to take very few. They were further told that people are frequently asked to make risky decisions for other people, not just for themselves such as when parents have to make risky decisions on behalf of their children and children are asked to make risky decisions on behalf of their parents. There are many other occasions and situations (such as at work or in a social context), where people make risky decisions. The goal of this part was to convey that taking risks is quite common but also that the willingness to take risks varies between people. Then, participants were told that in this study we are interested to test how people infer the degree to which another person is likely to engage in different risky activities.

Then, participants were informed that they will see a photograph depicting how an unknown person reacted to a specific event. Each participant saw and rated only one picture. They were asked to assume that the photo represents the person’s present state of mind. This was done to provide participants with a reason why the photos may include an emotional expression. Participants were then asked to look at the photo and rate the likelihood that this person would engage in different risky activities. For descriptions of risky activities or behaviors, we used the Domain-Specific Risk-Taking (DOSPERT) scale (Blais and Weber, 2006). This is the 30-item version of the DOSPERT scale, which is designed to evaluate behavioral intentions, or the likelihood with which respondents might engage in risky activities or behaviors originating from five domains (i.e., ethical, financial, health and safety, social, and recreational risks). Since the original scale measures a person’s own likelihood of engaging in each behavior, the scale was modified so that it referred to another person. Responses were made using a 7-point rating scale ranging from 1: Extremely Unlikely to 7: Extremely Likely. Item scores for each subscale were summed by adding up all items of a given subscale to obtain subscale scores. Thus, higher scores indicate a greater perceived willingness to take risks in a specific domain described by the items of the subscale. The full DOSPERT, in its original form, can be interpreted as a generalized risk propensity measure (Mishra and Lalumière, 2011). Accordingly, in the present context, it can be seen as reflecting the other person’s perceived generalized risk propensity. This measure was computed by averaging ratings across life domains.

Finally, as a manipulation check, we asked participants to rate the degree to which the person in the photo expressed anger, sadness, and neutrality. These ratings were also made on a 7-point rating scale ranging from 0: Not at all to 6: To a large extent.

The data from this study as well as from Study 2 are openly available in OSF at https://osf.io/nj6zt/?view_only=b78ad5411b4b4effa15dc2c5fee17d6e.

### Results and Discussion

#### Manipulation Check

A 3 (expressed emotion: anger, sadness, and neutral) × 2 (expresser gender) between-subjects analysis of variance was conducted on ratings of anger, sadness, and neutrality. The expected main effect of emotion expression was significant for all emotion conditions, anger: *F*(2,218) = 39.13, *p* < 0.001, *η_p_*^2^ = 0.26, sadness: *F*(2,218) = 59.33, *p* < 0.001, *η_p_*^2^ = 0.35, neutrality: *F*(2,218) = 60.49, *p* < 0.001, *η_p_*^2^ = 0.36. *Post-hoc* tests (*p* < 0.05) revealed that, for anger, displays of anger were rated as the angriest (*M* = 5.12, *SD* = 1.18, CI_95%_ 4.71; 5.53) and displays of sadness appeared as least angry (*M* = 2.76, *SD* = 2.11, CI_95%_ 2.31; 3.06) with displays of neutrality being perceived as reflecting an intermediate level of anger (*M* = 3.22, *SD* = 1.73, CI_95%_ 3; 3.73). Expressions of sadness were perceived as most sad (*M* = 4.75, *SD* = 1.49, CI_95%_ 4.39; 5.11) and expressions of anger as least sad (*M* = 1.82, *SD* = 1.62, CI_95%_ 1.43; 2.21) with neutral expressions perceived as showing an intermediate level of sadness (*M* = 3.37, *SD* = 1.68, CI_95%_ 3.01; 3.71). Finally, a neutral expression seemed most neutral (*M* = 3.65, *SD* = 1.67, CI_95%_ 3.3; 4) and expressions of anger (*M* = 1.3, *SD* = 1.65, CI_95%_ 0.92; 1.69) and sadness (*M* = 1.14, *SD* = 1.47, CI_95%_ 0.78; 1.51) were perceived similarly and as less neutral. Overall, as these results suggest, the expressions of emotions were perceived as intended.

#### Perceived Willingness to Engage in Risky Behaviors

First, we assessed the internal consistency of each of the subscales of the modified DSOPERT used in our study. Scores ranged from *α* = 0.68 to *α* = 0.86 (*α*_social_ = 0.68, *α*_recreational_ = 0.86, *α*_financial_ = 0.82, *α*_health/safety_ = 0.85, and, *α*_ethical_ = 0.84). Thus, overall, the scale showed adequate reliability. To test the effect of emotion expressions on perceived risk-taking likelihood, we conducted a series of two-way ANOVAs with emotion expression (anger, sadness, and neutral) and gender (men and women) as between-subjects factors for each risk domain separately, as well as for the combined measure reflecting generalized perceived risk propensity.

As shown in the first row of Table 1, the expected main effect of emotion was significant for the generalized perceived risk propensity as well as for all five risk domains. Across all ratings, as expected, anger led to an increased perception of risk proneness relative to sadness. In most life domains, except for the domains of ethics and health/safety, sadness led to less perceived risk proneness than neutrality. The risk proneness of people who expressed anger was in most cases rated similarly to that of people who showed neutrality. Only for the domain of ethics and the social domain, were ratings for anger higher than for neutrality.

**Table 1:** Perceptions of the willingness to take risks as a function of emotion expression for each risk domain – Study 1.

Risk domain	Anger *M* (*SD*) CI_95%_	Sadness *M* (*SD*) CI_95%_	Neutral *M* (*SD*) CI_95%_	*F*(2, 218)	*p*	η*_p_*^2^
Generalized risk propensity	27.42_a_ (4.54) 26.23; 28.61	22.54_b_ (5.77) 21.43; 23.65	25.97_a_ (4.60) 21.43; 23.65	18.95	<0.001	0.15
Ethics	29.18_a_ (6.25) 27.48; 30.89	24.86_b_ (7.84) 23.27; 26.45	24.34_b_ (6.82) 22.81; 25.87	10.03	<0.001	0.08
Finance	23.61_a_ (6.98) 21.90; 25.31	18.64_b_ (7.66) 17.05; 20.24	23.37_a_ (6.98) 21.90; 24.96	12.00	<0.001	0.10
Health/safety	29.88_a_ (7.59) 28.09; 31.67	26.34_b_ (8.33) 24.68; 28.01	27.60_ab_ (6.70) 26.02; 29.23	4.13	=0.017	0.04
Recreation	23.44_a_ (7.09) 21.68; 25.20	18.29_b_ (8.21) 16.65; 19.93	25.68_a_ (6.80) 24.15; 27.32	21.42	<0.001	0.16
Social	30.98_a_ (5.09) 29.71; 32.26	24.58_b_ (6.24) 23.39; 25.76	28.84_c_ (4.44) 27.72; 30.00	27.98	<0.001	0.20

Higher means reflect a higher level of perceived propensity to take risks. Within each row means marked by small letters differ significantly at least at *p* < 0.05.

As shown on Table 2 and as expected, for some of the risk domains as well as the generalized risk propensity, a gender effect emerged such that men were seen as more likely to engage in risks than women. This was not the case for the social and ethical domains. For the ethics domain there was no effect for gender; in the social domain, an interaction between gender and expressed emotions qualified these main effects, *F*(2,218) = 3.87, *p* = 0.02, η*_p_*^2^ = 0.03. Post-hoc tests (*p* < 0.05) revealed that whereas women seemed equally likely to take a social risk regardless of the emotion that they expressed (*M*_neutral_ = 28.07; *SD* = 4.17, CI_95%_ 26.48; 29.67, *M*_anger_ = 30.3; *SD* = 5.76, CI_95%_ 28.5; 32.1, and, *M*_sadness_ = 25.89; *SD* = 6.56, CI_95%_ 24.22; 27.57) men who expressed sadness (*M* = 23.26, *SD* = 5.69, CI_95%_ 21.59; 24.94) were perceived as less likely to take risks than men who expressed anger (*M* = 31.67; *SD* = 4.3, CI_95%_ 29.87; 33.47) or neutrality (*M* = 29.65; *SD* = 4.63, CI_95%_ 28.02; 31.47). Effects sizes for the emotion effect ranged from small to medium.

**Table 2:** Perceptions of the willingness to take risks as a function of target gender for each risk domain – Study 1.

Risk domain	Women *M (SD)* CI_95%_	Men *M (SD)* CI_95%_	*F*(1,218)	*p*	η*_p_*^2^
Generalized risk propensity	24.20 (5.39)	26.28 (5.18)	10.27	=0.002	0.05
Ethics	25.34 (6.82) 24.18; 26.81	26.56 (7.05) 25.44; 28.08	1.79	=0.18	0.01
Finance	20.19 (7.71) 18.93; 21.55	23.51 (7.01) 22.22; 24.86	12.24	=0.001	0.05
Health/safety	26.10 (7.57) 24.78; 27.53	29.62 (7.33) 28.36; 31.13	13.15	<0.001	0.06
Recreation	21.39 (7.62) 19.99; 22.69	23.66 (8.26) 22.27; 25.00	5.55	=0.019	0.03
Social	27.99 (5.76) 27.11; 29.07	28.06 (6.05) 27.21; 29.18	0.60	=0.88	0.00

Overall, these findings confirm that ratings of risk proneness were informed by incidental emotion expressions. Further, the effect of emotion expressions on the perception of risk proneness was not uniform across risk domains. This is congruent with findings for people’s own willingness to take risks (Soane and Chmiel, 2005; Hanoch et al., 2006; Figner and Weber, 2011). Notably, the significant effects of sadness and anger on perceived risk proneness, were always the same. Specifically, in line with our hypothesis, anger increased perceived willingness to take risks and sadness decreased it.

These findings are congruent with the notion that an action tendency of moving against, attacking, or removing an obstruction, signals to observers a willingness to also take risks other than the risk involved in moving against the other person. For sadness, the tendency to become inactive, seek help or recover, also seems to extend to a general tendency to be inactive and hence to not seek risks. However, the effect of sadness was more pervasive than the effect of anger as it had an impact on more risk domains than anger. In other words, it may be clearer to observers that someone who is inactive, or attempting to recover, is unlikely to engage in risky behavior. That someone who is inclined to attack or move against someone, may be taken as less indication that this inclination extends to other types of risks as well. In Study 2, we attempted to substantiate and extend these conclusions.

## Study 2

Study 1, is the first, to our knowledge, to demonstrate that observers consider others’ sadness and anger as informative when evaluating the emoter’s likelihood to engage in risky behavior in general as well as in specific domains of risk. In Study 2, we aimed to extend our findings by including disgust and happiness expressions along with expressions of sadness.

A growing body of research shows the significant role of disgust in determining risk-taking behaviors. This is based on the notion that disgust is an emotion that functions to regulate exposure to potential harm from contaminated resources. As such, it can be seen as a mechanism for risk avoidance related to contamination (Tybur and Lieberman, 2016; Sparks et al., 2018). Yet it has been suggested that disgust also functions to regulate decisions in the context of mate choice and morality (Tybur et al., 2013). As such, one may expect that expressions of disgust will be perceived as indicating a reduced willingness to take risks, in particular, those that are related to health and safety, ethics, and maybe also risks in the social domain.

The expressions examined in Study 1 were of negative emotions. However, positive emotions are also relevant in this context. Happiness is associated with a tendency or urge to play and broaden one’s experience (Fredrickson, 2004). Thus, a happy person may be seen as more inclined to afford taking risks. This may be more restricted to domains that match a happy state such as the recreational domain than the risk in domains that are likely to ruin the person’s happiness if the outcome is undesirable. Such may be the case in the domain of health or the financial domain. Accordingly, observers may assume that happy people are more likely to engage in risky behavior, at least relative to sad and disgusted people who in general are expected to avoid risks as detailed above. Nevertheless, based on our finding from Study 1, we expected sadness to decrease to a lesser degree the person’s perceived willingness to engage in risky behavior in the domains of health and safety, and ethics.

### Method

#### Participants

In total, 411 (216 women) participants with a mean age of 42 years (*SD* = 13.67) who were recruited through Amazon MTurk completed the study and passed control questions probing for attention. Based on a sensitivity analysis using G*Power (Faul et al., 2007), given our sample size, the minimum effect size that the experiment had 80% power to detect was *f* = 0.21.

#### Stimulus Materials and Procedure

Facial expressions of sadness, disgust, happiness, and neutrality by four young men and women were taken from the FACES database of facial expressions (Ebner et al., 2010). The procedure was the same as in Study 1 and hence will not be described here again. Measures were also the same as in Study 1, with the exception that for the manipulation check participants were asked, in addition, to rate perceived disgust and happiness. Since disgust is often confused with anger (see, e.g., Knutson, 1996; Du and Martinez, 2011), we included this rating scale in this study as well.

### Results and Discussion

#### Manipulation Check

A 4 (emotion expression: disgust, sadness, happiness, and neutral) X 2 (expresser gender) between-subjects analysis of variance was conducted on ratings of disgust, sadness, happiness, anger, and neutrality. A main effect of emotion expression emerged significantly for all emotion scales, disgust: *F*(3,403) = 146.25, *p* < 0.001, *η_p_*^2^ = 0.52, sadness*: F*(3,403) = 68.44, *p* < .001, η*_p_*^2^ = 0.34, happiness: *F*(3,403) = 173.38, *p* < 0.001, *η_p_*^2^ = 0.56, anger: *F*(3,403) = 49.88, *p* < 0.001, *η_p_*^2^ = 0.27, and neutrality: *F*(3,403) = 79.22, *p* < 0.001, *η_p_*^2^ = 0.37. Post-hoc tests (*p* < 0.05) revealed that, disgust expressions were rated as showing a greater degree of disgust (*M* = 5.3; *SD* = 0.8, CI_95%_ 5.02; 5.58) than all other expressions, with happiness showing less disgust (*M* = 1.02; *SD* = 1.55, CI_95%_ 0.73; 1.32) than sadness (*M* = 2.48; *SD* = 1.79, CI_95%_ 2.41; 3.05) or neutrality (*M* = 2.48; *SD* = 2.02, CI_95%_ 2.14; 2.8) which were rated similarly. Sad expressions were perceived as most sad (*M* = 4.51; *SD* = 1.58, CI_95%_ 4.15; 4.83); expressions of disgust (*M* = 2.24; *SD* = 1.88, CI_95%_ 1.94; 2.55) and neutrality (*M* = 2.61; *SD* = 1.76, CI_95%_ 2.23; 2.95) were rated similarly and as somewhat sadder than expressions of happiness (*M* = 1.11; *SD* = 1.55, CI_95%_ 0.79; 1.44). Expressions of happiness were rated as happiest (*M* = 5.26; *SD* = 0.88, CI_95%_ 4.97; 5.55) with neutrality being somewhat happier (*M* = 2.05; *SD* = 1.73, CI_95%_ 1.73; 2.37) than disgust (*M* = 1.25; *SD* = 1.68, CI_95%_ 0.97; 1.52) and sadness (*M* = 1.09; *SD* = 1.64, CI_95%_ 0.78; 1.39) which were rated similarly.

Recall that we also asked participants to rate the expressions on perceived anger since disgust is often seen as also reflecting anger to some degree. Indeed, expressions of disgust were rated as angrier (*M* = 4.04; *SD* = 1.56, CI_95%_ 3.73; 4.36) than all other expressions with happiness seeming least angry (*M* = 1.24; *SD* = 1.67, CI_95%_ 0.92; 1.58) and sadness (*M* = 2.27; *SD* = 1.81, CI_95%_ 1.94; 2.64) and neutrality (*M* = 2.56; *SD* = 1.81, CI_95%_ 2.2; 2.93) rated equally in-between. Finally, neutral expressions were rated as most neutral (*M* = 4.06; *SD* = 1.61, CI_95%_ 1.17; 1.76) with sadness (*M* = 1.15; *SD* = 1.68, CI_95%_ 0.91; 1.53) rated similarly to disgust (*M* = 0.98; *SD* = 1.40, CI_95%_ 0.7; 1.26); happiness (*M* = 1.47; *SD* = 1.54, CI_95%_ 1.17; 1.76) was seen as somewhat more neutral than disgust. A significant main effect also emerged for gender, *F*(1,403) = 4.31, *p* = 0.039, *η_p_*^2^ = 0.01, such that women seemed somewhat sadder (*M* = 2.7; *SD* = 2, CI_95%_ 2.54; 3.03) then men (*M* = 2.44; *SD* = 2.16, CI_95%_ 2.21; 2.66). Overall, all expressions were perceived as intended. Further, as expected, expressions of disgust were perceived as reflecting a moderate level of anger.

#### Perceived Willingness to Engage in Risky Behaviors

As in Study 1, we first assessed the internal consistency of each of the subscales of the modified DSOPERT that we used in our study. Scores ranged from *α* = 0.71 to *α* = 0.86 (*α*_social_ = 0.71, *α*_recreational_ = 0.86, *α*_financial_ = 0.8, *α*_health/safety_ = 0.83, and, *α*_ethical_ = 0.84, for the social, recreational, financial, health and safety, and ethical domains, respectively). Thus, the scale showed adequate reliability.

**Figure 1:**
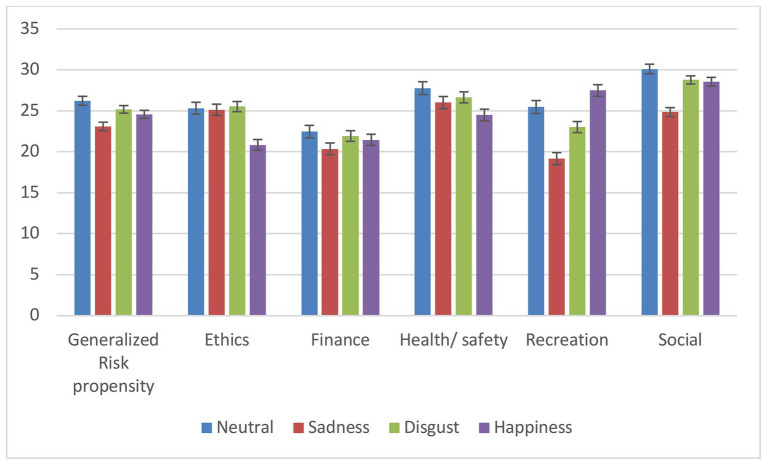
Means and standard errors for perceptions of willingness to take risks as a function of emotion expression for each risk domain – Study 2.

As for Study 1, we conducted a series of two-way ANOVAs with emotion expression (disgust, sadness, happiness, and neutral) and gender (male and female) as between-subjects factors and ratings of perceived generalized risk propensity and risk-taking in each risk domain as dependent variables. As shown in Figure 1 and Table 3, a main effect of emotions emerged for the generalized risk propensity and for all risk domains except for the financial domain. For generalized risk, post-hoc tests (*p* < 0.05, see Table 3), showed that we replicated the findings from Study 1 in that participants perceived the sad person as less inclined to take risks relative to persons in any other condition. The happy person was judged to be more inclined to take risks than the neutral person but not more than the disgusted person. The latter conditions were not significantly different.

**Table 3:** Perceptions of the willingness to take risks as a function of emotion expression for each risk domain – Study 2.

Risk domain	Disgust *M* (*SD*) CI_95%_	Sadness *M* (*SD*) CI_95%_	Happiness *M* (*SD*) CI_95%_	Neutral *M* (*SD*) CI_95%_	*F*(3, 403)	*p*	η*_p_*^2^
Generalized risk propensity	25.18_ac_ (4.87) 24.26; 26.05	23.10_b_ (5.58) 21.97; 23.98	24.57_c_ (5.21) 23.56; 25.44	26.23_a_ (4.36) 25.25; 27.35	7.18	<0.001	0.05
Ethics	25.52_a_ (7.06) 24.28; 26.73	25.13_a_ (6.56) 23.63; 26.38	20.85_b_ (6.74) 19.50; 22.08	25.32_a_ (6.63) 23.89; 26.76	11.65	<0.001	0.08
Finance	21.93 (6.99) 20.66; 23.14	20.36 (7.02) 18.66; 21.43	21.46 (7.21) 20.03; 22.63	22.47 (6.91) 21.17; 24.06	2.34	=0.07	0.02
Health/safety	26.65_a_ (7.21) 25.33; 27.92	26.01_ab_ (7.06) 24.34; 27.24	24.49_b_ (7.72) 22.92; 25.64	27.77_a_ (7.12) 26.36; 29.38	4.33	=0.005	0.03
Recreation	23.02_a_ (7.55) 21.67; 24.28	19.16_b_ (8.47) 17.63; 20.54	27.48_c_ (6.29) 26.08; 28.82	25.47_c_ (6.85) 24.05; 27.09	24.93	<0.001	0.16
Social	28.77_a_ (4.90) 27.79; 29.76	24.84_b_ (6.79) 23.84; 26.05	28.56_a_ (5.07) 27.60; 29.68	30.11_a_ (4.82) 28.96; 31.26	15.42	<0.001	0.10

Higher means reflect a higher level of perceived propensity to take risks. Within each row, means marked by small letters differ significantly at least at *p* < 0.05.

Turning to the ratings of perceived risk-taking willingness in each risk domain, as in Study 1, expressions of sadness made the person seem least likely to engage in risky behavior in the social and recreational domains. On the other hand, in Study 1 for the social domain, this was the case only for men; in Study 2, this was the case for both men and women.

However, unlike in Study 1, sadness did not have an effect on the perceived likelihood to engage in risks in the financial domain. This was the case for all emotional expressions in this study. Given this generalization of all emotions, we suspect that at the time we conducted the study, some real-world event suggested that this was a bad time to take financial risks and that this information overrode the effect of the emotional expression.

Disgust reduced perceived risk proneness relative to neutrality only for the recreational domain. For all other domains, ratings were similar to those in the neutral expression condition. That disgust did not have an effect on health and safety risks seems to contradict the notion that the function of disgust is to avoid contamination (Tybur et al., 2013; Tybur and Lieberman, 2016; Sparks et al., 2018). However, the health and safety related scale items were not related to risks associated with contamination. Rather, they were about risking one’s health or safety by using illegal drugs, excessive alcohol drinking, engaging in unprotected sex, not wearing a seatbelt while being in a car, or a helmet when riding a motorcycle, or sun exposure without sunscreen. Also, as noted above, disgust expressions were also rated as showing considerable anger, and in Study 1 anger expressions also had no impact on perceived risk-taking likelihood in the health and safety domain relative to neutrality. Further, the failure to find an effect of disgust for the ethical domain speaks against its possible function in this context as suggested by Tybur et al. (2013).

However, expressions of disgust reduced perceived risk proneness in the recreational domain relative to neutrality and happiness (but not sadness, which was still lower). This could suggest a general tendency to see people who experience a negative emotion as less likely to engage in recreational activity to the extent that they take risks.

In turn, emoters who showed happiness were considered to be more likely to take risks in the recreational domain relative to sadness and disgust but not relative to neutrality. Further, happiness was the only expression that reduced perceived willingness to take risks in the ethical domain. One reason could be that happy people are perceived as unmotivated to take risks that can hurt them and ruin their mood, as suggested by research that examined the link between felt happiness and risk-taking behavior (Isen and Geva, 1987). Finally, expressions of happiness reduced the perceived willingness to take risks in the health and safety domain relative to neutrality and disgust but not relative to sadness.

Next, we examined whether women and men were perceived differently in terms of willingness to take risks, in general, as well as in specific life domains. In this context, the effects were as expected and similar to those found in Study 1. That is, women, overall, were judged to be less likely to take risks than men were. This was also true when looking at specific risk domains except for the ethical and social domains in which men and women were seen as equally likely to take risks (see Table 4).

**Table 4:** Perceptions of the willingness to take risks as a function of target gender for each risk domain – Study 2.

Risk domain	Women *M* (*SD*) CI_95%_	Men *M* (*SD*) CI_95%_	*F*(1,403)	*p*	η*_p_*^2^
Generalized risk propensity	23.84 (4.74) 23.07; 24.5	25.53 (5.35) 25.01; 26.34	14.55	<0.001	0.04
Ethics	23.69 (7.12) 22.71; 24.65	24.56 (6.94) 23.72; 25.54	1.98	=0.16	0.01
Finance	19.74 (6.89) 18.70; 20.66	23.11 (6.84) 22.35; 24.18	27.50	<0.001	0.06
Health/safety	24.56 (7.03) 23.54; 25.59	27.56 (7.37) 26.76; 28.68	19.45	<0.001	0.05
Recreation	22.59 (7.75) 21.50; 23.56	24.84 (7.55) 24.05; 25.98	11.90	=0.001	0.03
Social	28.61 (5.22) 27.69; 29.25	27.58 (6.12) 27.03; 28.49	1.69	=0.19	0.00

In sum, as expected and as in Study 1, emotion expressions were used as cues for observers’ estimates of the likelihood that the expressers will engage in risky behavior. In particular, we replicated the finding that sadness overall reduces perceived risk proneness. At the same time, as expected, specific emotion effects varied between risk domains.

However, not all expected effects were found, especially with regard to disgust expressions. Disgust only reduced perceived risk proneness in the recreational domain. Especially the failure of disgust expressions to impact on perceived risk proneness with regard to health and safety, and the ethical domain is puzzling as the hypothesized core functions of disgust relate to these domains as noted above.

One possible reason may lie in the stimuli themselves. In fact, the disgust expressions we used also signaled anger. This is not uncommon even for pretested disgust expressions (e.g., Du and Martinez, 2011). That the effects of disgust expressions on perceived risk proneness largely matched the results for anger expressions, as found in Study 1, supports this possibility. As such, the anger component in these expressions may, at least to some degree, obscure the unique effect of disgust expressions. In fact, so-called secondary emotions, emotions perceived in addition to the target emotion, have been shown to affect observers’ perceptions in meaningful ways (Hess et al., 2016; Hareli et al., 2018).

To explore the contribution of anger versus disgust perceptions to perceptions of potential risk-taking, we conducted an exploratory analysis using perceived emotion ratings instead of the emotion expression condition. For this, ratings of perceived disgust, anger, sadness, happiness, and neutrality were used as predictors of the perceived willingness to engage in risky behavior in each of the life domains. For this analysis, we used the lm function of R (R Core Team, 2018) and lm.beta (Behrendt, 2014). As can be seen in Table 5, the results are very similar to the results of the previous analyses, but they allow us to assess the differential effect of perceived disgust and anger. In addition, this analysis provides a better sense of the direction of the effect of each type of expression on perceived risk willingness for each risk domain.

**Table 5:** Summary of multivariate multiple regression analysis of perceived expressions predicting perceived risk-taking willingness in each risk domain.

Risk domain	Ethics					Finance					Health and Safety				
Emotion	*B*	*SE B*	*β*	*t*(405)	*p*	*B*	*SE B*	*β*	*t*(405)	*p*	*B*	*SE B*	*β*	*t*(405)	*p*
Disgust	0.42	0.22	0.13	1.89	=0.06	0.10	0.24	0.03	0.40	=0.69	0.31	0.24	0.12	1.26	=0.21
Sadness	0.24	0.16	0.06	1.45	=0.15	−0.07	0.18	−0.02	−0.42	=0.68	−0.04	0.18	−0.01	−0.23	=0.82
Happiness	−0.26	0.16	−0.08	−1.68	=0.09	0.24	0.17	0.09	1.40	=0.16	0.08	0.17	0.03	0.45	=0.65
Anger	0.85	0.24	0.30	3.57	<0.001	0.55	0.26	0.16	2.16	=0.03	0.95	0.26	0.24	3.65	<0.001
Neutral	0.55	0.17	0.15	3.33	<0.001	0.71	0.18	0.20	3.97	<0.001	0.48	0.18	0.13	2.62	=0.009
	*F*(5,405) = 19.67, *p* < 0.001					*F*(5,405) = 6.71, *p* < 0.001					*F*(5,405) = 11.62, *p* < 0.001				
*R*^2^	0.20					0.08					0.13				
Risk domain	Recreation					Social									
Emotion	*B*	*SE B*	*β*	*t*(405)	*p*	*B*	*SE B*	*β*	*t*(405)	*p*					
Disgust	0.26	0.25	0.08	1.06	=0.29	0.39	0.19	0.12	2.07	<0.05					
Sadness	−0.74	0.18	−0.27	−4.08	<0.001	−0.51	0.14	−0.15	−3.70	<0.001					
Happiness	1.06	0.17	0.35	6.09	<0.001	0.45	0.13	0.13	3.40	<0.001					
Anger	0.11	0.27	0.03	0.42	=0.68	0.43	0.20	0.12	2.15	<0.05					
Neutral	0.93	0.19	0.23	5.03	<0.001	0.56	0.14	0.19	4.04	<0.001					
	*F*(5,405) = 22.65, *p* < 0.001					*F*(5,405) = 14.32, *p* < 0.001									
*R*^2^	22					0.15									

First, perceived anger had a stronger impact on the perceived likelihood to engage in risky behavior than perceived disgust. This is true for all domains except for the recreational domain for which both anger and disgust had no effect. Further, perceived disgust had a unique effect on perceived risk taking in the social domain and to some degree in the ethical domain. In addition, the direction of this effect was identical to the effect of perceived anger, namely, a higher degree of perceived disgust and or anger increased the perceived likelihood of the person to engage in risky behavior in these domains. As such, despite the theoretical function of disgust to avert risky behavior in these domains (Tybur et al., 2013; Tybur and Lieberman, 2016; Sparks et al., 2018), this was not what observers concluded. If anything, the reverse was the case. Finally, this analysis also suggests that the effect of perceived happiness is mostly in the direction of increasing perceived risk taking except for the domain of ethics. What also stands out from this analysis is that neutrality contributes quite substantially and positively to the perceived likelihood of taking risks.

Overall, our data from Study 2 revealed that emotional expressions are perceived by observers as indicative of the willingness of the expresser to engage in risky behavior. More importantly, we were able to demonstrate that the effect of specific expressions of emotions on perceived willingness to engage in risky behavior is not uniform across different risk domains.

## General Discussion

Emotion expressions signal the likely behaviors of the emoter (Frijda, 1987; Frijda et al., 1989; Roseman et al., 1994; Fontaine et al., 2007; Scherer and Grandjean, 2008). Much of the research on perceived action tendencies has focused on abstract actions that are not related to a specific context. By contrast, in this research, we show that observers also use this information when considering behavior in a specific context even when this behavior does not directly represent the action tendency. Specifically, we investigated whether observers would translate the general behavioral tendencies associated with specific emotions into aspects of risk-taking behavior.

Whereas prior research has considered how the naïve knowledge of the relation between emotion antecedent events and the resulting emotion is used by observers to make sense of emoters and their situation (Hareli and Hess, 2010, 2019; de Melo et al., 2014; Hareli, 2014;), little is known about the use of emotion-specific actions tendencies to predict specific behaviors. In the present research, we examined how (and whether) expressions of emotions influence judgments of the expressers’ willingness to engage in risky behavior. We predicted that specific emotions, because they are associated with specific behaviors in people’s naïve theories, can be used to estimate how willing a person would be to take risks.

In line with this idea, our findings demonstrated that observers judge others’ risk proneness on the basis of incidental emotions that others express. In two studies we found that emotion expressions had a systematic impact on observers’ ratings of the expressers’ risk proneness. This was the case both for general risk proneness and for risk proneness in specific domains. Further, we found that the effect was not uniform across different risk domains. In particular, expressions of anger resulted in higher perceived risk proneness in the ethical and social domains. By contrast, sadness reduced perceived risk proneness in the financial domain (Study 1 only), as well as the recreational and social domains. Happiness expressions reduced perceived risk proneness in the ethical and health and safety domains relative to neutrality but did not increase it relative to neutrality in the recreational domain. Finally, disgust expressions had a relatively limited unique effect on risk perceptions that was limited to social risks.

A regression analysis with perceived emotions as predictors indicated that perceived neutrality has a strong impact on the perception of risk proneness. As such, this adds to the notion that neutrality is not the simple absence of emotions and does not convey any message (Carrera-Levillain and Fernandez-Dols, 1994). In the present context, neutrality can be conceived of as a state of “coolness.” That is the person is calm and has no worries and hence may be more likely to engage in risky behavior.

In the present study, we manipulated emotional expression by showing participants photos of standardized facial expressions of emotions. However, we do not assume that the results are specific to facial expressions. Rather we assume that the results readily generalize to situations where emotions are expressed vocally or posturally or where emotion information is provided verbally by using a label.

Doctors, lawyers, parents, and employers are often called upon to make (crucial) decisions for their patients, children, clients, and prospective employees. The information they integrate into their decision-making when they have to estimate others’ willingness to take risks is of key importance. Research so far has focused on stereotype information (Siegrist et al., 2002) and the self (Hsee and Weber, 1997) as anchors for such judgments as well as for the accuracy of such judgments (Roth and Voskort, 2014), and have largely failed to examine the role emotions might play in the process. Our findings clearly indicate that a person’s emotional expression is used when judging their willingness to take risks, and furthermore, that the judgment also depends on the risk domain. This extends previous research on the cues that observers can use to understand how a person may behave in a given context.

## Data Availability Statement

The datasets presented in this study can be found in online repositories. The names of the repository/repositories and accession number(s) can be found at: https://osf.io/nj6zt/?view_only=b78ad5411b4b4effa15dc2c5fee17d6e.

## Ethics Statement

The studies involving human participants were reviewed and approved by the Ethics Committee of the Faculty of Social Science University of Haifa (#217/20). The patients/participants provided their written informed consent to participate in this study.

## Author Contributions

SH and YH contributed to conception and design of the study. SE organized the database and programmed the studies. UH performed the statistical analysis. SH wrote the first draft of the manuscript. UH, YH, and SE wrote the sections of the manuscript. All authors contributed to manuscript revision, read, and approved the submitted version.

### Conflict of Interest

The authors declare that the research was conducted in the absence of any commercial or financial relationships that could be construed as a potential conflict of interest.
